# The application of NIPT using combinatorial probe-anchor synthesis to identify sex chromosomal aneuploidies (SCAs) in a cohort of 570 pregnancies

**DOI:** 10.1186/s13039-018-0407-z

**Published:** 2018-12-03

**Authors:** Hongge Li, Yu Lei, Hui Zhu, Yuqin Luo, Yeqing Qian, Min Chen, Yixi Sun, Kai Yan, Yanmei Yang, Bei Liu, Liya Wang, Yingzhi Huang, Junjie Hu, Jianyun Xu, Minyue Dong

**Affiliations:** 1 Women’s Hospital, School of Medicine Zhejiang University, 1, Xueshi Road, Hangzhou Zhejiang, 310006 People’s Republic of China; 2 Key Laboratory of Reproductive Genetics (Zhejiang University), Ministry of Education, 1, Xueshi Road, Hangzhou Zhejiang, 310006 People’s Republic of China; 3 Key Laboratory of Women’s Reproductive Health of Zhejiang Province, 1, Xueshi Road, Hangzhou Zhejiang, 310006 People’s Republic of China

**Keywords:** Non-invasive prenatal testing (NIPT), Sex chromosomal aneuploidies (SCAs), Combinatorial probe-anchor synthesis (CPAS)

## Abstract

**Background:**

Non-invasive prenatal testing (NIPT) as alternative screening method had been proven to have very high sensitivity and specificity for detecting common aneuploidies such as T21, T18, and T13, with low false positive and false negative rates. Unfortunately, recent studies suggested that the NIPT achieved lower accuracy in sex chromosomal aneuploidies (SCAs) detection than autosomal aneuploidies detection. BGISEQ-500 powered by Combinatorial Probe-Anchor Synthesis (CPAS) and DNA Nanoballs (DNBs) technology that combined linear amplification and rolling circle replication to reduce the error rate while enhancing the signal. Therefore, NIPT based on CPAS might be a good method for SCAs screening in routine clinical practice. In the study, we intended to evaluate the clinical utility of NIPT based on CPAS on screening for fetal SCAs.

**Results:**

A total of 570 pregnant women were included in the retrospective study. Maternal blood samples were collected for NIPT; amniocentesis was performed on all pregnant women. NIPT was carried out by BGISEQ-500 sequencing platform based on CPAS. Karyotype analysis of amniotic cells was performed by standard G-banding techniques. 43 out of the total 570 pregnant women tested by NIPT showed fetal SCAs (19 of 45,X, 12 of 47,XXY, 10 of 47,XXX, and 2 of 47,XYY). The following amniocentesis confirmed that 26 cases were true positive (7 of true positive 45,X, 9 of true positive 47,XXY, 9 of true positive 47,XXX as well as 1 of 47,XYY) and the positive predictive value (PPV) for fetal SCAs was 60.47%. In addition, the PPV of advanced maternal age group (67.74%) was higher than the other indications group (45.45%) or serological screening high-risk /critical-risk group (0%).

**Conclusions:**

NIPT based on CPAS could be a potential method for SCAs screening. However, it still had high false positive rates, especially for 45,X. The pregnant women with fetal SCAs detected by NIPT, especially those with non-age-related prenatal diagnostic indications, should be advised to accept invasive prenatal karyotype analysis.

## Background

Since cell-free fetal DNA (cffDNA) was detected in cell-free DNA (cfDNA) of pregnant women plasma by Lo et al. in 1997 [1], comparison of chromosome dosage distribution of cffDNA between patient and control plays an increasingly important role in fetal aneuploidy diagnosis [2]. NIPT calculated the risk of fetal chromosomal aneuploidies by detecting cffDNA circulating in maternal plasma with the next-generation sequencing technology [3]. The NIPT as alternative screening method had been proven to have > 99% sensitivity and a false-positive rate of < 0.1% for autosomal aneuploidies such as trisomy 21 (T21, Down syndrome), trisomy 18 (T18), trisomy 13 (T13) [4]. Unfortunately, recent studies suggested that the NIPT achieved lower accuracy in SCAs detection than autosomal aneuploidies detection [5, 6]. One of the most important reasons is that SCAs detection is highly dependent on fetus sex prediction [2]. SCAs such as Turner (45,X), Klinefelter (47,XXY), Triple X syndrome (47,XXX) and Jacob syndromes (47,XYY) affected approximately one in 1000 births [7], early interventions such as hormone therapy and hormone replacement therapy had been shown to improve the outcomes for babies with 45,X syndrome or 47,XXY syndrome [8]. So prenatal screening and prenatal diagnosis about SCAs were critical for outcome preparation and timely treatment [9].

NIPT had been rapidly used in clinical in China since 2011 [10]. The Women’s Hospital School of Medicine, Zhejiang University had validated and implemented NIPT successfully on October 2012, and obtained the first batch of National Clinical Trial Unit qualification in 2015. BGISEQ-500 is a recently launched high-throughput sequencing solution, powered by Combinatorial Probe-Anchor Synthesis (CPAS) and improved DNA Nanoballs (DNBs) technology. The CPAS chemistry works by incorporating a fluorescent probe to a DNA anchor on the DNB, followed by high-resolution digital imaging. This combination of linear amplification and DNB technology reduces the error rate while enhancing the signal [11–13]. Therefore, NIPT based on CPAS might be a good method for SCAs screening in routine clinical practice. In the study, we intended to evaluate the clinical utility of NIPT based on CPAS on screening for fetal SCAs.

## Materials and methods

### Subjects

A total of 570 pregnant women who visited the Women’s Hospital School of Medicine, Zhejiang University for prenatal counseling from May 2017 to October 2017 were included in the retrospective study. The study was carried out under the approvement by the Hospital Ethics Committee of the Women’s Hospital School of Medicine, Zhejiang University. All 570 pregnant women had received amniocentesis and karyotype analyzing at Women’s Hospital School of Medicine, Zhejiang University.

The indications for NIPT were as follows:

Maternal serological screening high-risk value (T21 ≥ 1/270, T18 ≥ 1/350) or critical-risk value (1/1000 ≤ T21 < 1/270, 1/1000 ≤ T18 < 1/350), advanced maternal age (35 years or older at the expected date of confinement), abnormal fetal structure identified by ultrasound, adverse pregnancy history or pregnant women involving “anxiety” who voluntarily required NIPT.

The exclusion criteria were in accordance with the rules of Chinese government and were as follows: twin or multiple pregnant women, either parent of the fetus with chromosomal abnormality, pregnant women who undergo embryo transplantation or stem cell therapy, and pregnant women who had a history of immunotherapy within 1 month or allogeneic blood transfusion within 1 year.

Among the 570 pregnant women analyzed by NIPT, 49 were found to have autosomal aneuploidies, including 3 of T13 (3/570), 8 of T18 (8/570) and 38 of T21 (38/570). 43 were found to have SCAs (43/570), including 19 of 45,X (19/570), 12 of 47,XXY (12/570), 10 of 47,XXX (10/570), and 2 of 47,XYY (2/570).

### Collection and treatment of blood samples

Maternal peripheral blood samples (5 ml) were collected in EDTA tubes, fully mixed, stored temporarily in 4 °C refrigerator. Samples were excluded if hemolysis or storage beyond 8 hours before plasma separation. The blood sample was treated as follows: centrifuged at 4 °C, 1600 g for 10 min and the plasma was collected carefully and dispensed into 2.0 ml Eppendorf tubes. The plasma was centrifuged again at 4 °C, 16,000 g for another 10 min. The upper plasma was carefully divided into new 2.0 ml Eppendorf tubes and each contained approximately 600 ml plasma, − 80 °C refrigerator to save. The plasma should avoid repeated freezing and thawing before experiment.

### Non-invasive prenatal testing (NIPT)

The DNA extraction, library construction, and sequencing were performed according to the standard protocol of the Women’s Hospital School of Medicine, Zhejiang University of Human Molecular Genetics Guidelines. 200 μl maternal plasma was used for cffDNA extraction by BGISP-300 (BGI, Shenzhen, China) with Nucleic Acid Extraction Kit (BGI, Shenzhen, China). The next step after DNA extraction was end-repair by adding end-repair enzymes with the following cycle conditions: 37 °C for 10 min and 65 °C for 15 min, then followed by adaptor ligation at 23 °C for 20 min with label-adaptor and ligase. After end-repair and adaptor ligation, PCR was used to amplify DNA to desired concentration with the following cycle conditions: 98 °C for 2 min, then 12 cycles at 98 °C for 15 s, 56 °C for 15 s, and 72 °C for 30 s, with a final extension at 72 °C for 5 min. The DNA amplification products were quantified by Qubit® 2.0 (Life Tech, Invitrogen, USA) using QubitTM dsDNA HS Assay Kits (Life Tech, Invitrogen, USA) and the concentration ≥ 2 ng/μl was regarded as qualified standards. The volume was calculated according to the concentration of each sample, and each sample of the same mass was mixed by pooling. The DNA double strands were thermally denatured into single strand after pooling, and then the cyclic buffer and the ligase were added to make DNA circles by the cyclization reaction. DNA circles were used to make DNBs by rolling circle replication (RCR). The concentration of DNBs was quantified by Qubit® 2.0 using QubitTM ssDNA Assay Kits (Life Tech, Invitrogen, USA) and the DNBs concentration in the range of 8 ng/μl-40 ng/μl was considered as appropriate concentration. The DNBs were loaded onto chips and sequenced on BGISEQ-500 sequencing platform (BGI, Shenzhen, China). Fetal chromosome aneuploidies (T21, T18, and T13) detection kit (Combinatorial Probe-Anchor Synthesis Sequencing Method) (BGI, Shenzhen, China) was used for library construction and Sequencing was performed using Universal Reaction Kit for Sequencing (Combinatorial Probe-Anchor Synthesis Sequencing Method) (BGI, Shenzhen, China). Any sample that failed to meet the quality control criteria was reported as detection failure by NIPT. Z-score set the range of − 3 and 3 as the threshold to evaluate the risk of chromosomal aneuploidies. If Z-score > 3 or Z-score < − 3, the sample was classified as high-risk of chromosomal aneuploidies. If Z-score was between − 3 to 3, the sample was classified as low risk.

### Amniocentesis and fetal karyotyping

Amniocentesis was performed at gestational age of 19^+ 0^ to 22^+ 6^ weeks. All pregnant women signed informed consents before the invasive procedure. Amniocentesis was performed by the certificated physicians under ultrasound guidance. The initial 2 ml of amniotic fluid was discarded and the other 20 ml of amniotic fluid was collected for karyotype analysis. Amniotic fluid cells were cultured according to the standard protocol of the Women’s Hospital School of Medicine, Zhejiang University of Human Cytogenetics Guidelines. Chromosome analysis with targeted 400-band level were performed and at least 30 metaphases were counted and 5 metaphases were analyzed for each sample by using GSL-120 high-throughput automatic chromosome scanning platform (Leica, German) after G-banding. If there were different cell lineages in the same patient, the count was increased to 50–100 metaphases to establish the mosaicism. Chromosomal abnormalities were described according to the criteria established by the International System for Human Cytogenetic Nomenclature (ISCN, 2016).

### Statistics

The statistics were calculated treating the invasive procedure results as the gold standard, which included the false positive rate (FPR), false negative rate (FNR), sensitivity (SEV), specificity (SPC) as well as positive predict value (PPV) and negative predict value (NPV) in the study. The 95% confidence intervals (CI) were calculated on the bases of a standard normal distribution by Clopper-Pearson method.

## Results

In the present study, a total of 570 pregnant women were enrolled for the NIPT trial according to different prenatal diagnosis indications, including 328 with advanced maternal age, 78 with serological screening high-risk, 2 with serological screening critical-risk, and 162 with other indications (ultrasound fetal structural abnormalities or ultrasound soft indexes abnormality, adverse pregnancy history and pregnant women who voluntarily required NIPT). The proportion of the different prenatal diagnosis indications was listed in Table 1.

**Table 1:** The prenatal diagnostic indications of 570 pregnant women

Prenatal diagnostic indications	Cases(%)
Advanced maternal age	328 (57.54%)
Serological screening high-risk value	78 (13.68%)
Serological screening critical-risk value	2 (0.35%)
Other indications	162 (28.42%)
Total	570 (100.00%)

After NIPT detection, 43 pregnant women had abnormal SCAs results as follows: 45,X in 19/570; 47,XXY in 12/570; 47,XXX in 10/570 and 47,XYY in 2/570. The following amniocentesis results confirmed that there were 26 cases of true SCAs (26/570), including 7 true positive of 45,X (7/570), 9 true positive of 47,XXY (9/570), as well as 9 true positive of 47,XXX (9/570) and one of 47,XYY (1/570). All 49 pregnant women (T13 in 3/570, T18 in 8/570 and T21 in 38/570) with autosomal aneuploidies detected by NIPT had the same results with the amniocentesis. The SCAs results of NIPT and amniocentesis were listed in Table 2. Further analysis showed that the overall FPR of SCAs in the study was 3.13%, the FPR of 45,X, 47,XXY, 47,XXX, and 47,XYY was 2.13, 0.53, 0.18, and 0.18%, respectively. The number of false negative was 0, and the FNR of SCAs in the study was 0% (Table 3).

**Table 2:** The result comparison between NIPT and amniocentesis for fetal sex chromosomal abnormalities

Case	Age	NIPT result	Amniocentesis result
1	40	45,X	45,X
2	39	45,X	45,X
3	36	45,X	45,X[46]/46,X,i(X)(q12) [4]
4	32	45,X	45,X[48]/46,X,i(Y)(q10) [2]
5	37	45,X	45,X [7]/46,XX[93]
6	38	45,X	45,X [11]/46,XX[39]
7	25	45,X	45,X [1]/46,XX[99]
8	41	45,X	46,N,t (X;1)(p22;p23)
9	36	45,X	46,X,der(X)
10	37	45,X	46,X,i(Xq)
11	30	45,X	47,XXX
12	34	45,X	Normal
13	39	45,X	Normal
14	29	45,X	Normal
15	28	45,X	Normal
16	35	45,X	Normal
17	29	45,X	Normal
18	34	45,X	Normal
19	39	45,X	Normal
20	42	47,XXX	47,XXX
21	26	47,XXX	47,XXX
22	44	47,XXX	47,XXX
23	40	47,XXX	47,XXX
24	38	47,XXX	47,XXX
25	42	47,XXX	47,XXX
26	38	47,XXX	47,XXX
27	23	47,XXX	47,XXX
28	35	47,XXX	47,XXX
29	33	47,XXX	45,X
30	35	47,XXY	47,XXY
31	35	47,XXY	47,XXY
32	40	47,XXY	47,XXY
33	38	47,XXY	47,XXY
34	39	47,XXY	47,XXY
35	36	47,XXY	47,XXY
36	36	47,XXY	47,XXY
37	25	47,XXY	47,XXY
38	44	47,XXY	47,XXY,inv.(9)
39	43	47,XXY	47,XYY
40	39	47,XXY	47,XYY[40]/46,XX [12]
41	37	47,XXY	Normal
42	40	47,XYY	47,XYY
43	35	47,XYY	47,XXY

**Table 3:** The FPR and FNR of fetal SCAs based on BGISEQ-500 sequencing platform

Types	NIPT high-risk(case)	TP(case)	FP(case)	TN (case)	FN (case)	FPR (%)	FNR (%)
45,X	19	7	12	551	0	2.13%	0%
47,XXY	12	9	3	558	0	0.53%	0%
47,XXX	10	9	1	560	0	0.18%	0%
47,XYY	2	1	1	568	0	0.18%	0%
Total	43	26	17	527	0	3.13%	0%

*TP* true positive, *FP* false positive, *TN* true negative, *FN* false negative, *FPR* false positive rate, *FNR* false negative rate

The SEV, SPC, NPV, and PPV of SCAs by NIPT based on BGISEQ-500 were listed in Table 4. The overall SEV was 100% (95% CI: 86.77–100.00%), SPC was 96.88% (95% CI: 95.04–98.17%) and NPV was 100%. The overall PPV was 60.47% (95% CI: 48.93–70.95%), and the PPV for 45,X, 47,XXY, 47,XXX, and 47,XYY was 36.84% (95% CI: 25.00–50.52%), 75.00% (95% CI: 49.25–90.27%), 90.00% (95% CI: 55.95–98.46%%), and 50.00% (95% CI: 12.37–87.63%), respectively.

**Table 4:** The SEV, SPC, PPV and NPV of NIPT for fetal SCAs based on BGISEQ-500 sequencing platform

	45,X	47,XXY	47,XXX	47,XYY	Total
NIPT high-risk(case)	19	12	10	2	43
TP (case)	7	9	9	1	26
FP (case)	12	3	1	1	17
TN(case)	551	558	560	568	527
FN (case)	0	0	0	0	0
SEV (%)	100%	100%	100%	100%	100%
(95%CI)	(59.04–100.00%)	(66.37–100.00%)	(66.37–100.00%)	(2.50–100.00%)	(86.77–100.00%)
SPC (%)	97.87%	99.47%	99.82%	99.82%	96.88%
(95%CI)	(96.31–98.89%)	(98.45–99.89%)	(99.01–100.00%)	(99.02–100.00%)	(95.04–98.17%)
PPV(%)	36.84%	75.00%	90%	50%	60.47%
(95%CI)	(25.00–50.52%)	(49.25–90.27%)	(55.95–98.46%)	(12.37–87.63%)	(48.93–70.95%)
NPV(%)	100%	100%	100%	100%	100%
Accuracy(%)	97.89%	99.47%	97.89%	99.82%	97.02%
(95%CI)	96.35–98.91%	98.47–99.89%	96.35–98.91%	99.03–100.00%	95.27–98.25%

*TP* true positive, *FP* false positive, *TN* true negative, *FN* false negative, *SEV* sensitivity, *SPC* specificity, *PPV* Positive predict value, *NPV* Negative predict value, *CI* Confidence interval

We further classified the 43 pregnant women with fetal SCAs according to different prenatal diagnostic indications (Table 5). 31 patients were from advanced maternal age group, one patient was from serological screening high-risk / critical-risk group and 11 patients from other indications group. The true positive rate of SCAs in advanced maternal age group was 67.74%, and the positive rate of 45,X, 47,XXY, 47,XYY, and 47,XXX in advanced maternal age group was 45.45, 72.73, 50, and 100%, respectively. And the overall positive rate in other group was 45.45%, and the positive rate of 45,X, 47,XXY, 47,XXX in other indications group was 25, 100, 100%, respectively. The positive rate of SCAs in serological screening high-risk / critical-risk value group was 0%.

**Table 5:** The PPV of NIPT for fetal SCAs with different prenatal diagnosis indications

Sex chromosomal aneuploidies	Prenatal diagnostic indications		
	True positive rate(%)(Amniocentesis/ NIPT)		
Types	advanced maternal age	others	serological screening high/ critical risk value
45,X	45.45(5/11)	25.00(2/8)	–
47,XXY	72.73(8/11)	100.00(1/1)	–
47,XYY	50.00 (1/2)	— (0/0)	–
47,XXX	100.00(7/7)	100.00(2/2)	0.00(0/1)
Total	67.74(21/31)	45.45(5/11)	0.00(0/1)

## Discussion

Massive Parallel Genome Sequencing (MPGS) (also called as next-generation sequencing, NGS) had been widely used in NIPT for years [5, 14]. NIPT as a successful application in routine clinical practice has been widely used to detect trisomy 21, 18, 13 and SCAs [15, 16]. The result of fetal sex prediction was dependent on SCAs detection. However, the current data processing method has some limitations to achieve higher accuracy. In the present study, we investigated the clinical utility of NIPT based on CPAS that applied DNBs technology for sequencing library construction and CPAS for massively parallel DNA sequencing, and found that NIPT based on BGISEQ-500 sequencing platform was suitable for SCAs detection.

There were 43 pregnant women with fetal SCAs shown by NIPT, and the overall PPV was 60.47% (95%CI 48.93–70.95%), which was higher than some existing researches [17–19]. The higher PPV reflected a lower omission diagnose rate and a higher detection rate of target disease based on BGISEQ-500 sequencing platform. The source of the patient might be an important factor. As the largest obstetrics and gynecology hospital in Zhejiang Province, the majority of patients were relatively higher risk individuals. In addition, the small number of samples tested might also be an important contributing factor. For example, there were only two cases of 47,XYY, which would result in wide 95% CI (12.37–87.63%) and low statistical power. The PPV range from 47,XXX (90, 95%CI 55.95–98.46%), 47,XXY (75.00, 95%CI 49.25–90.27%), 47,XYY (50, 95%CI 12.37–87.63%) to 45,X (36.84, 95%CI 25.00–50.52%). The PPV for detecting 45,X was the lowest, indicating the NIPT based on BGISEQ-500 sequencing platform for 45,X still had high false positive rates and the accuracy needed to be further improved. However, the PPV for detecting 45,X was 36.84%, which was relative higher compared with other studies [19]. Therefore, NIPT that based on BGISEQ-500 sequencing platform for screening fetal SCAs still need more improvement. Based on our study, the prevalence of true SCAs in population was 4.56% (26/570), which would avoid 95.46% of unnecessary invasive procedures. However, compared with amniocentesis, NIPT based on the BGISEQ-500 sequencing platform for SCAs still had high false positive rates, especially for Turner’s syndrome. Therefore, the pregnant women with fetal SCAs by NIPT should be advised to accept invasive prenatal karyotype analysis.

At present, the NIPT platform was highly sensitive and specific for screening autosomal trisomy [20, 21]. However, the application of prenatal diagnosis for SCAs was still in constant exploration [21, 22]. According to our results, the false positive rate and the false negative rate for SCAs was 3.13 and 0%, respectively. In addition to the limitations of noninvasive screening techniques, the source of cffDNA is another important factor to lead to inconsistent results between NIPT and traditional invasive diagnosis. It was well known that the presence of cffDNA circulating in maternal plasma was derived from the placental trophoblast [23]. These discordant cases are predominantly due to confined placental mosaicism (CPM). CPM can lead to false positive (abnormal cells in placenta but normal in fetus); or false negative results (abnormal cells in the fetus but not placenta) [24]. In addition, true fetal mosaicism can also lead to discordant NIPT results, which has been predicted to occur in 1/107 cases [24]. Maternal etiology for discordant results can arise if the mother is a mosaic herself, this is particularly the case when using NIPT to screen for SCAs [25].

In the study, the sensitivity and negative predict value of all kinds of SCAs were 100%, suggesting that NIPT can identify more positive cases and can effectively reduce the chance of misdiagnosis in population. The comparisons of specificity are as follows: 47,XYY (99.82, 95%CI 99.02–100.00%) = 47,XXX (99.82, 95%CI 99.01–100.00%) > 47,XXY (99.47, 95%CI 98.45–99.89%) > 45,X (97.87, 95%CI 96.31–98.89%). The overall sensitivity and specificity were 100% (95%CI 86.77–100.00%) and 96.88% (95% CI 95.04–98.17%), which was relative higher compared with some existing researches [26, 27]. This indicated that the BGISEQ-500 sequencing platform had certain advantages in detecting SCAs, but whether the BGISEQ-500 sequencing platform was superior to other sequencing platforms still needed more discussion. The comparisons of accuarcy are as follows: 47,XYY (99.82, 95%CI 99.03–100.00%) > 47,XXY (99.47, 95% CI 98.47–99.89%) > 47,XXX (97.89, 95%CI 96.35–98.91%) = 45,X (97.89, 95%CI 96.35–98.91%), the high accuracy provided wide application prospect in the field of scientific research and clinical testing.

This finding makes us think further about the relationship between the specificity and the PPV of NIPT analyses. The PPV of NIPT is the highest for T21 and is much lower for other aneuploidies [28–32]. PPV depends not only on the sensitivity and specificity of the assay, but also on the prevalence of the disease [33]. In addition, our study was designed to compare the results of NIPT screening and karyotype analysis for fetal SCAs only in patients who had results from both tests and not to compare the performance in actual clinical practice. We excluded the “no call” results including some cases who did not complete NIPT screening or patients without karyotype results. The prevalence was based on evaluating 570 pregnant women in this manuscript, the small numbers in this cohort prevented an accurate assessment of the sensitivity and PPV of the methods.

Due to the relatively low amount of cffDNA, fetal fraction of Cell-Free DNA is a prerequisite for successful NIPT testing. Low fetal fraction was another cause of discordant inconsistent results between NIPT and traditional invasive diagnosis, and higher concentration of fetal DNA associated with more accurate aneuploidy results [34]. The method of sample collection is important for maintaining fetal fraction. More cffDNA is found in the plasma of maternal blood instead of the cellular fraction [35]. It is important to maintain maternal cells integrity when collecting samples for cfDNA extraction. Inappropriate sample collection leads to reduced the detection of fetal fraction. If using of K3EDTA tube sample should be processed within 8 h, otherwise cell-stabilizing tubes should be used [36]. A total of 570 plasma samples were analyzed by NIPT in the study and none of them occurred hemolysis or placed for more than 8 h before treatments. Among these 570 pregnant women, 328 (57.54%) were due to advanced maternal age without other prenatal diagnostic indications and the other 242 (42.46%) were in appropriate age (aged under 35 years at delivery). Among the 242 pregnant women, 80 (14.04%) of them were maternal serological screening high-risk / critical-risk, and 162 (28.42%) of them were abnormal ultrasound diagnosis or undesirable reproductive history. Among the 43 pregnant women with fetal SCAs, 31 were due to advanced maternal age without other prenatal diagnostic indications. The PPV of advanced maternal age group (67.74%) was higher than that of other indication group (45.45%) and serological screening high-risk / critical-risk group (0%), which was consistent with the PPV of 45,X. At the same time, we could figure out that the PPV of 45,X in advanced maternal age group (45.45%) and other prenatal diagnostic indications group (25.00%) were lower than that of other sex chromosomal abnormalities, which was consistent with the previous studies [37, 38], however, we could not draw a credible conclusion because of fewer subjects in other sex chromosomal abnormality group. Of the 43 cases, only one of them was due to high-risk value of serological screening and none due to critical-risk value of serological screening, indicating that although a large number of pregnant women accepted NIPT because of serological screening high-risk / critical-risk, the incidence of fetal SCAs was relatively low.

## Conclusions

NIPT based on CPAS could be a potential method for SCAs screening, while this technique needed to be further investigated. It had high false positive rates, especially for 45,X. The pregnant women with fetal SCAs by NIPT, especially those with non-age-related prenatal diagnostic indications, should be advised to accept invasive prenatal karyotype analysis.

## Abbreviations

cfDNA: Cell-free DNA
cffDNA: Cell-free fetal DNA
CI: Confidence intervals
CPAS: Combinatorial Probe-Anchor Synthesis
DNBs: DNA Nanoballs
FNR: False negative rate
FPR: False positive rate
HRT: Hormone replacement therapy
ISCN: International System for Human Cytogenetic Nomenclature
NGS: Next-generation sequencing
NIPT: Non-invasive prenatal testing
NPV: Negative predict value
PPV: Positive predictive value
RCR: Rolling circle replication
SCAs: Sex chromosomal aneuploidies
SEV: Sensitivity
SPC: Specificity
TPR: True positive rates
